# Supplementary material for: Super-Resolution Fluorescence Microscopy Reveals Clustering Behaviour of Chlamydia pneumoniae’s Major Outer Membrane Protein
Source: Biology (Basel). 2020 Oct 20;9(10):344. doi: 10.3390/biology9100344 (PMC7589890; doi:10.3390/biology9100344)
Supplement: Supplementary file 1 [file biology-09-00344-s001.pdf]

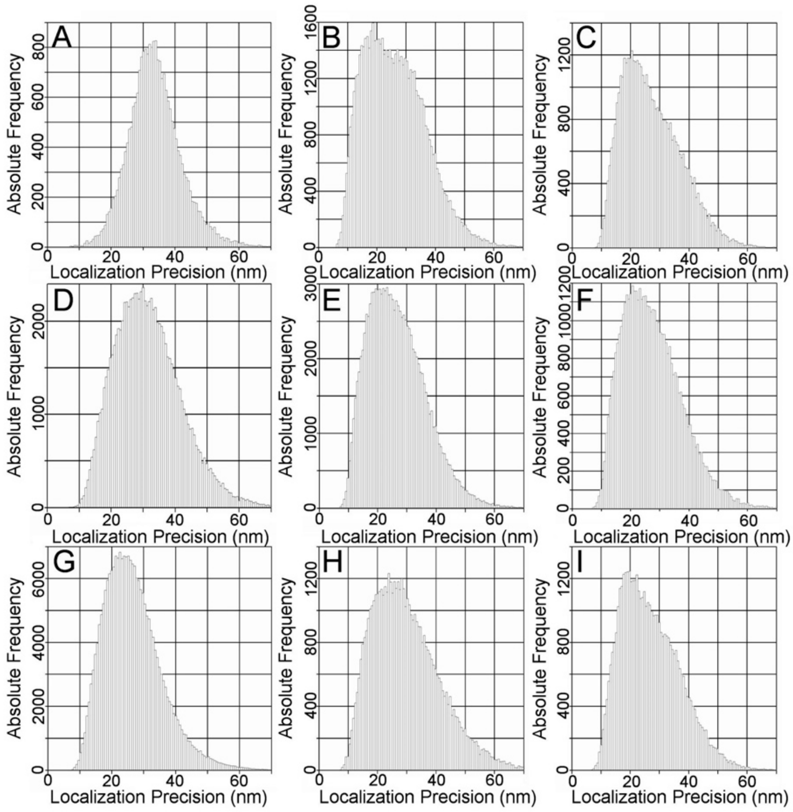

Figure S1. Localisation precision histograms corresponding to dSTORM data from Figure 3. **A)** *E. coli* anti-OmpA; **B)** wild type rMOMP; **C)** rMOMP C201A; **D)** rMOMP C203A; **E)** rMOMP C201/203A; **F)** rMOMP C136A; **G)** rMOMP C136/201A; **H)** rMOMP C136/203A; and **I)** rMOMP C226A.

Table S1. Primers used for cysteine mutant production. Bold indicates the location of the cysteine mutation and underline indicates a cysteine previously mutated.

Mutation	Forward	Reverse
C136A	GGGATCGCTTTGATGTTTTC**GCT**ACTTTAGGAGCTTCTAATGG	CCATTAGAAGCTCCTAAAGT**AGC**GAAAACATCAAAGCGATCCC
C201A	CTCGTGGAGCCTTATGGGAA**GCC**GGTTGTGCAACTTTG	CAAAGTTGCACAACC**GGC**TTCCCATAAGGCTCCACGAG
C203A	GCCTTATGGGAATGCGGT**GCT**GCAACTTTGGGAG	CTCCCAAAGTTGC**AGC**ACCGCATTCCCATAAGGC
C201A/C203A	GCCTTATGGGAA<u>GCC</u>GGT**GCT**GCAACTTTGGGAG	CTCCCAAAGTTGC**AGC**ACCGGCTTCCCATAAGGC
C226A	GTTGAAGAACTTAATGTGATC**GCT**AACGTATCGCAATTCTCTGTAAAC	GTTTACAGAGAATTGCGATACGTT**AGC**GATCACATTAAGTTCTTCAAC
